# Comparing the Efficacy of Digital and In-Person Weight Loss Interventions for Patients with Obesity and Glycemic Disorders: Evidence from a Randomized Non-Inferiority Trial

**DOI:** 10.3390/nu16101510

**Published:** 2024-05-16

**Authors:** Katarína Moravcová, Markéta Sovová, Jaromír Ožana, Martina Karbanová, Jan Klásek, Agnieszka Barbara Kolasińska, Eliška Sovová

**Affiliations:** 1 Department of Exercise Medicine and Cardiovascular Rehabilitation, University Hospital Olomouc, I. P. Pavlova 6, 779 00 Olomouc, Czech Republic; marketa.sovova@fnol.cz (M.S.); jaromir.ozana@fnol.cz (J.O.); eliska.sovova@fnol.cz (E.S.); 2 Faculty of Medicine, Palacký University Olomouc, Hněvotínská 3, 775 15 Olomouc, Czech Republic; 3 Department of Public Health, Masaryk University, Kamenice 5, 625 00 Brno, Czech Republic; martina.karbanova@vitad.io; 4 First Faculty of Medicine, Charles University in Prague, Kateřinská 32, 121 08 Prague, Czech Republic; 5 Vitadio s.r.o., Římská 678/26, 120 00 Prague, Czech Republic; jan.klasek@vitad.io (J.K.); agnieszka.kolasinska@vitad.io (A.B.K.)

**Keywords:** obesity, digital therapeutics, lifestyle intervention, diabetes mellitus type 2, insulin resistance

## Abstract

Digital weight loss interventions present a viable and cost-effective alternative to traditional therapy. However, further evidence is needed to establish the equal effectiveness of both approaches. This randomized controlled non-inferiority trial aimed to compare the effects of an intensive in-person weight loss intervention program with Vitadio digital therapy. One hundred patients with obesity and diagnosed with type 2 diabetes, prediabetes, or insulin resistance were enrolled and randomly assigned to one of the two treatment groups. Over a 6-month period, the control group received five in-person consultations with a physician who specialized in obesity treatment, a dietitian and/or a nutrition nurse, while the intervention group followed the digital program based on a multimodal therapeutic approach. The extent of weight loss was assessed and compared between the groups. Additionally, changes in body composition and metabolic parameters for the digital intervention group were analyzed. The study results demonstrated comparable effectiveness of both treatments for weight reduction. The positive effects of Vitadio were further evidenced by favorable changes in body composition and lipid metabolism and improved glycemic control in the intervention group. These findings suggest that Vitadio is an effective tool for assisting patients with managing obesity and preventing diabetes progression.

## 1. Introduction

Digital therapeutics (DTx) is a relatively new term referring to a sub-category of digital health solutions that deliver evidence-based interventions aimed at preventing, managing, or treating a medical condition or disease through the use of qualified software programs [1]. They can be used independently or in combination with medication or other forms of traditional therapies. A distinctive characteristic of DTx is that the software itself, rather than the hardware it is installed on, is classified as a medical device [2]. As they can be easily deployed on a standard smartphone, DTx have the potential of becoming widely accessible to a broad population.

Digital therapeutics may improve patients’ outcomes by promoting lifestyle changes, enabling continuous monitoring of health parameters, and improving the efficacy of proven therapies, e.g., by enhancing patient adherence to medication [3]. Thus, they are particularly relevant for the treatment of chronic conditions such as obesity [4].

Obesity is a complex, multifaceted problem stemming from a combination of genetics, lifestyle choices, and societal, cultural and psychological factors [5,6]. Excessive weight is known to increase the risk of various diseases, such as cardiovascular diseases, diabetes and cancers [7,8,9,10,11]. Therefore, addressing obesity through prevention and treatment is crucial for reducing the chances of developing chronic conditions, making it one of the key public health priorities [5,12].

However, obesity management is a challenging process that requires a multidisciplinary and multimodal approach integrating aspects of nutrition, physical activity, behavior change and sometimes pharmacotherapy [6,13] or bariatric surgery. Given the significant burden it places both on the patients and their healthcare providers, there is a growing demand for innovative treatment strategies that can support the weight loss journey. Research has shown that incorporating mobile technology such as personal digital assistants and mobile apps into standard obesity treatments can enhance patients’ short-term weight loss outcomes, providing a scalable mechanism to augment physician-directed treatment [14]. This approach holds potential for reaching a broader patient population at a reduced cost compared to traditional in-person care.

At the same time, addressing obesity with mobile health applications poses some challenges. High attrition rates from weight management programs are a well-recognized problem in patients with obesity [15]. Some studies report dropout rates as high as 80% [16]. While mHealth interventions offer greater flexibility and accessibility of care, enabling the circumvention of some of the obstacles associated with conventional weight loss programs, factors such as technological literacy, personal motivation, and intervention engagement play significant roles in participant retention. Therefore, the primary challenge in designing DTx lies in ensuring the engaging nature of the therapeutic product and its suitability for diverse groups of patients, including older adults who may be less comfortable with using new technologies [17].

In this article, we present the final results of a study aiming to compare the effectiveness of an intensive in-person weight reduction program and an evidence-based digital lifestyle intervention—Vitadio. We expected the two treatments to yield similar positive results (non-inferior). Sensitivity analysis was conducted using an intention-to-treat (ITT) approach to address concerns about potential bias introduced by a substantial amount of missing data. Additionally, we examine the impact of Vitadio on anthropometric measures and clinical outcomes such as glucose and lipid metabolism as well as liver function parameters to provide a comprehensive overview of the intervention’s health-related effects. Lastly, we assess users’ satisfaction with the Vitadio app and attempt to shed some light on the factors that influence participants’ treatment adherence.

## 2. Materials and Methods

### 2.1. Study Design

Preliminary results of this prospective, double-armed, randomized non-inferiority trial along with a detailed description of the study design and methods have already been reported elsewhere [18]. In this paper, we present the final six months of data of the study and aim to evaluate whether the digital lifestyle intervention yields results comparable to those of an intensive personalized weight reduction program in patients with obesity. The trial has been registered at https://www.clinicaltrials.gov/study/NCT04573296?term=NCT04573296&rank=1, accessed on 1 March 2022, (NCT04573296) and was approved by the Ethics Committee of the University Hospital Olomouc (ref. number 10/20) in January 2020.

### 2.2. Participants

One hundred participants were included in the study following an eligibility assessment performed at the Department of Exercise Medicine and Cardiovascular Rehabilitation, University Hospital Olomouc, between February 2020 and November 2021. Patients were screened during routine clinical practice and were considered to be eligible for inclusion if they were 18 or older, had a body mass index (BMI) > 30 kg/m^2^ and were diagnosed with one of the following conditions: type 2 diabetes mellitus or prediabetes (characterized by fasting glucose between 5.6–6.9 mmol/L or oral glucose tolerance test (OGTT) results falling within the range of 7.8–11.0 mmol/L) or insulin resistance (IR) (defined as Homeostatic Model Assessment for Insulin Resistance (HOMA-IR) > 2.7). Additionally, all participants were expected to have a smartphone compatible with the Vitadio application and to be able and willing to comply with the study procedures.

Participants were considered not eligible to join the study based on the following exclusion criteria: pregnancy, steroid treatment, severe renal or hepatic disease, insulin therapy, above the age of 60, and inability to comprehend and comply with the study procedures or to use the Vitadio app.

### 2.3. Study Procedures

After being screened for eligibility, participants were randomly assigned to either the intervention group (IG) or the control group (CG). Following the group assignment, a baseline visit was carried out to collect clinical and demographic data from each participant. The laboratory and anthropometric tests were conducted again during the three- and six-month follow-up visits. A participant was considered to have successfully completed the study when they had attended all the foreseen follow-up visits. Patients did not receive any financial or non-financial compensation for their participation in the study.

#### 2.3.1. Intervention Group

Vitadio is a certified class I medical device designed to support diabetes patients in making healthy lifestyle choices and improving their self-management. The digital care program is based on a multimodal therapeutic approach and consists of a 3-month intensive phase followed by a sustaining phase. The application uses a series of personalized daily tasks and automated messages to help patients establish a new, healthy routine.

Patients have access to an engaging educational course built around topics such as diet, physical exercise, motivation, sleep quality, mental and social health and well-being. Lessons are implemented using gamified personal goals that help patients root important habits into their daily lives. Patients are also encouraged to regularly monitor their physiological and lifestyle parameters. In order to guarantee patients’ safety and ensure that they capitalize fully on the proposed intervention, a qualified dietitian was available on the in-app chat to answer participants’ queries or concerns. There was also an option to have an informational call at the beginning of the study. Finally, a peer support group was available within the app to provide patients with a safe space for sharing their experiences and struggles.

#### 2.3.2. Control Group

Over the course of the study, the control group was offered access to five in-person lifestyle consultations with a physician, dietitian and/or educational nurse with a nutrition background from the Department of Exercise Medicine and Cardiovascular Rehabilitation. During the appointment, patients were offered recommendations on healthy eating habits. They were advised against restrictive weight-loss diets and instructed to follow balanced eating plans based on a high intake of fruits and vegetables, healthy fats, whole grains and lean proteins. Additionally, an online food diary, which contained recipes, exemplary menus, an energy intake calculator and macronutrient analysis, was offered to the participants.

### 2.4. Outcome Measures

The aim of this study was to compare the effectiveness of Vitadio and an intensive in-person weight reduction program for obesity management. Therefore, the primary outcome of this study was weight change from baseline to the end of the 6-month intervention period. The margin for evaluating whether the digital intervention was at least as effective as the in-person intervention was specified as a 3% body weight reduction, which is considered the minimal weight reduction associated with clinical benefits (for glycemic measures and triacylglycerols) [19].

In order to provide a comprehensive overview of the effects of Vitadio on health outcomes, exploratory subgroup analyses of changes in the anthropometric parameters (BMI, body composition and waist circumference) and laboratory outcomes (glucose and lipid metabolism and liver function parameters) for the intervention group were performed. Our focus was on exploring the additional potential benefits of the digital care program rather than proving its non-inferiority in relation to the secondary health outcomes. Therefore, we present the within-group analysis exclusively for the intervention group without comparison to the control group.

To assess users’ satisfaction with Vitadio and improve our understanding of the attrition patterns, we analyzed progress forms available within the app and baseline differences between those who completed the study and those who dropped out.

### 2.5. Statistical Analysis

Baseline and in-app data were summarized using standard descriptive statistics, including the mean and standard deviation for continuous variables and the frequency of occurrence, presented as absolute numbers and percentages, for categorical variables.

Clinical results were assessed using two separate datasets. A complete case (CC) analysis was conducted on patients who attended the 6-month study visit as specified in the trial protocol. The differences in the sample sizes for different outcome measures are due to missing data caused by protocol deviations (e.g., participants had their anthropometric measurements recorded, but they did not show up for the laboratory tests). Furthermore, an intention-to-treat (ITT) analysis was conducted. Under the missing-at-random (MAR) assumption, missing data for the ITT analysis were imputed using a reference-based multiple imputation approach, as suggested by Carpenter et al. [20], using the R package *RefBasedMI.* Given the study design (active comparator group), we applied the last mean carried forward (LMCF) method for all the investigated variables. This method was selected based on the assumption that the treatment effect persists beyond participant drop-out. Each dataset was imputed 20 times, matching an approximate percentage of missing data [21].

For the analysis of the primary outcome—weight—a linear mixed-effects model is employed, accounting for unobserved patient-to-patient differences as the random effect. Explanatory variables (fixed effects) include the visit number, treatment group, age, sex, highest achieved education, the diabetes progression stage and the interaction between the visit number and treatment. The interaction term captures the treatment effect of interest, which is the change in weight at follow-up visits. Pooled results of the linear mixed effect models are reported using robust standard errors. Additionally, the non-inferiority of Vitadio is evaluated with a pre-specified margin of 3% using Student’s *t*-test. Secondary outcomes are reported for IG only as mean differences between the baseline and 6-month endpoint of the study using Student’s *t*-tests for continuous variables; effect sizes are calculated using Cohen’s d and are presented together with the 95% CI.

Throughout the study, *p*-values less than or equal to 0.05 were considered statistically significant. All analyses were done using R software (version 4.3.2).

## 3. Results

### 3.1. Participant Flow and Baseline Characteristics

Participants’ demographic characteristics, baseline anthropometric measurements and biochemical parameters are presented in Table 1. There were no significant differences between the two groups at the beginning of the study. Each group comprised more women (IG: 68%; CG: 74%) than men. The average age of the sample was 43.3 ± 9.5.

**Figure 1:**
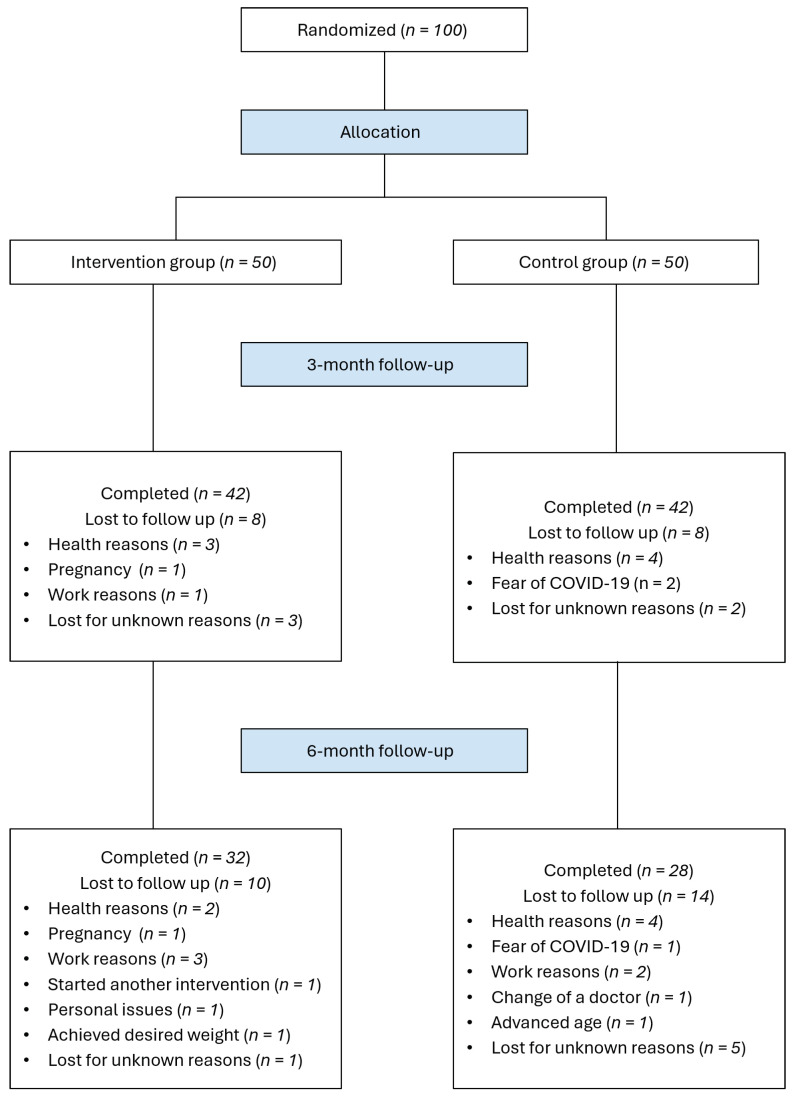
Study flow diagram depicting the progression of participants throughout the trial.

Of the 100 participants recruited for the study, 60 completed both the 3-month and 6-month follow-up visits. Overall attrition was higher in the control group (IG: 36%; CG: 44%). The most frequent reasons for study withdrawal were health issues (13%), work-related commitments (6%) and fear of exposure to COVID-19 (3%). Figure 1 shows the participant flowchart, including reasons for exclusions.

Anthropometric measurements were available for all the participants who completed the study at all time points. Laboratory tests were characterized by some missing values derived from protocol deviations.

### 3.2. Primary Outcome

#### 3.2.1. Complete Case Analysis

The complete case analysis of weight change over a six-month duration comprised 32 participants in the IG and 28 participants in the CG, respectively. Table 2 describes significant weight reduction in both groups over the observed period. The intervention group experienced an average weight decrease of −7.25 kg (*p* < 0.001), whereas the CG’s average weight loss was −8.32 kg (*p* < 0.001). Linear mixed model analysis suggested a significant time effect on the weight change (*p* < 0.001); no significant interaction was found between group assignment and time, highlighting the comparability of weight loss efficacy between groups, where, despite higher weight loss in the control group, the 3% non-inferiority margin is satisfied (*p* = 0.008).

#### 3.2.2. ITT Analysis

Analysis of the ITT sample has shown less-pronounced yet still significant and meaningful weight decrease in both groups: −6.28 kg (*p* < 0.001) in the IG and −5.21 kg (*p* < 0.001) in the CG (see Table 3). The outcomes of the linear mixed model show a significant time effect (−6.28 kg, *p* < 0.001) and non-significantly higher weight loss for the IG by 1.07 kg (*p* = 0.56). Notably, while the extent of the difference between groups is coincidentally similar, using the ITT data suggests better outcomes for the IG in contrast to the results suggested by the complete case analysis. This discrepancy suggests the different nature of dropouts across groups. The results provide additional evidence of the non-inferiority of the outcomes of Vitadio (*p* < 0.03).

### 3.3. Secondary Outcomes

Changes in anthropometric and body composition measures, glucose and lipid metabolism, and liver function parameters in the IG over the 6-month period are presented in Table 4.

#### 3.3.1. Complete Case Analysis

For the complete case analysis of the secondary outcomes, further participants were excluded when secondary endpoint data were not available at 6 months. Therefore, the CC sample included 32 participants for the anthropometric measures, 24 participants for parameters of glucose metabolism, 25 participants for the lipid parameters and 22 participants for the liver enzymes.

After the 6-month intervention, individuals in the intervention group experienced a significant decrease in waist circumference (−6.12 ± 5.63 cm, *p* < 0.001) and BMI (−2.54 ± 2.09 kg/m^2^, *p* < 0.001). Subsequent examination of changes in body composition showed a successful reduction in body fat (−6.97 ± 6.75 kg, *p* < 0.001) with no loss of lean muscle (0 ± 2.35 kg, *p* = 1.0).

The results of analysis of the changes in glucose metabolism observed within the IG from baseline to the 6-month follow-up indicate a significant reduction of glycated hemoglobin (HbA1c) levels (−0.38 ± 0.68%, *p* = 0.013) with no statistically significant changes observed in other glycemic parameters. Although there were no significant differences in fasting glucose levels, further analysis showed that 26% of the participants lowered their blood glucose levels below the threshold of 5.6 mmol/L for impaired fasting glucose.

The results indicate positive effects of Vitadio on the reduction of total cholesterol (−0.34 ± 0.6 mmol/L, *p* = 0.008) and triacylglycerols (−0.83 ± 0.98 mmol/L, *p* < 0.001). Moreover, a significant increase was observed in high-density lipoprotein (HDL) cholesterol (0.08 ± 0.15 mmol/L, *p* = 0.017). The change in low-density lipoprotein (LDL) cholesterol was not statistically significant.

There were no significant changes in the levels of liver enzymes following the intervention period.

#### 3.3.2. ITT Analysis

The results of ITT analysis for the secondary outcomes confirm the positive effects of Vitadio on waist circumference reduction (−5.12 ± 6.29 cm, *p* < 0.001), BMI lowering (−2.1 ± 2.29 kg/m^2^, *p* < 0.001) and body fat loss (−5.97 ± 6.74 kg, *p* < 0.001). Notably, alterations in muscle mass remained insignificant (*p* = 0.931).

All the positive changes in the lipid profiles remained significant in the ITT analysis, showcasing a decrease in total cholesterol (−0.16 ± 0.51 mmol/L, *p* = 0.032) and triacylglycerols (−0.5 ± 0.86 mmol/L, *p* < 0.001) coupled with a notable increase in HDL-C (0.06 ± 0.12 mmol/L, *p* < 0.001).

Furthermore, analysis of the ITT sample shows less-pronounced yet still significant changes in HbA1c from the baseline to the 6-month follow-up (−0.18 ± 0.55%, *p* = 0.029).

No other changes in the secondary outcomes reached statistical significance in the ITT analysis.

### 3.4. User Satisfaction Data

Participants in the IG were administered voluntary in-app questionnaires to assess their satisfaction with the treatment after 3 and 6 months; all answers were provided on a scale from 1 (lowest) to 10 (highest). App users were asked how satisfied they were with the Vitadio program (−8.43 ± 1.91 (*n* = 21) after 3 months; 8.12 ± 1.93 (*n* = 16) after 6 months), and whether they would recommend Vitadio to someone like them (−9.62 ± 1.16 (*n* = 21) after 3 months; not administered after 6 months).

### 3.5. Factors Associated with Attrition

Out of all participants assigned to the IG, 18 (36%) dropped out and did not complete the final 6-month assessment. To investigate potential factors contributing to attrition, we compared the baseline characteristics of individuals who completed the study with those who dropped out (see Table 5). There were no significant differences between the two groups in terms of initial weight (*p* = 0.21), BMI (*p* = 0.181) or weight circumference (*p* = 0.725). Participants who successfully completed the study were slightly older than those who withdrew (46.12 ± 9.32 vs. 38.28 ± 10.94), with this age difference being statistically significant (*p* = 0.015).

## 4. Discussion

The aim of this non-inferiority trial was to compare the effectiveness of Vitadio—a digitally administered lifestyle intervention—with an intensive weight reduction program. To account for the relatively high attrition rate, known to be one of the most challenging aspects of randomized controlled trials (RCTs) for weight loss and obesity [16], we performed sensitivity analysis based on the intention-to-treat principle. Overall, the results of both the primary analysis and sensitivity checks confirmed our hypothesis that both treatments were equally effective for weight reduction within the predefined boundaries of non-inferiority (3%). This is in line with the results of a recent systematic review [22] indicating that digital interventions for weight loss are comparable to face-to-face interventions. These results have important practical implications, as digital health technologies have the potential to improve the accessibility of weight management programs. They may help to circumvent the most frequently reported barriers to effective obesity management—limited time and resources of physicians—and the costs incurred by patients following the standard treatment [23,24].

The analysis of secondary outcomes provides further evidence of the effectiveness of Vitadio for obesity management. Participants within the intervention group managed to significantly reduce their BMIs. While weight loss is believed to be a crucial component of lowering the risk of obesity-related comorbidities, there is mounting evidence suggesting the importance of careful examination of other concomitant factors, such as changes in body composition and obesity phenotypes [25,26,27]. The obesity phenotype that poses the greatest health risk is excessive accumulation of abdominal fat [10,28,29]. Evidence suggests that waist circumference predicts the likelihood of developing diabetes mellitus beyond that explained by BMI alone [10,30], and its reduction is an essential part of the treatment of metabolic syndrome [31]. The digital intervention employed in the present study resulted in a significant reduction in abdominal obesity, represented mainly by changes in waist circumference, and overall body fat. Moreover, the observed weight loss was not associated with a loss of lean body mass. This is important since preserving lean body mass is not only essential for weight loss sustainability [32] but is also known to be a protective factor for cardiovascular diseases and is associated with greater insulin sensitivity [33,34].

Moreover, the intervention group achieved favorable changes in their lipid profiles. They successfully reduced the levels of total cholesterol and triacylglycerols while simultaneously increasing the values of HDL cholesterol. Notably, following the intervention period, the average concentration of triacylglycerols decreased to levels within the recommended healthy range of less than 1.7 mmol/L [35]. This holds particular importance considering the growing recognition of the necessity to control for the “residual risk” in individuals who have attained their LDL cholesterol goals [36]. In the present study, the baseline values of LDL-C in the intervention group were near optimal. However, while LDL-C is a well-documented contributor to atherosclerosis and cardiovascular diseases (CVDs), evidence suggests that patients may still face cardiovascular events despite lowering LDL levels effectively [37]. Thus, evaluating non-high-density lipoprotein (non-HDL) cholesterol levels—total cholesterol minus HDL cholesterol—might be more reliable than LDL-C alone for predicting CVD risk [38].

Due to the high representation of participants with insulin resistance in the analyzed sample (*n* = 12) and their near-optimal baseline HbA1c values, the interpretation of the intervention’s effects on glycemic control needs to be done with caution. Nonetheless, it should be noted that during the study, Vitadio users experienced a significant decrease in HbA1c levels, and 26% of them achieved a fasting glucose reduction below the 5.6 mmol/L threshold. These findings suggest that the digital lifestyle intervention may effectively enhance glycemic control, even in prediabetic patients.

Upon analysis of the attrition data, it was found that the only factor associated with study drop-out was younger age. Although the studied sample comprised mainly middle-aged individuals and lacked data from older adults, the noted tendency may indicate that the older population is more likely to adhere to the proposed treatment. That would align with the underlying principles of the intervention, which was designed to meet the needs of individuals aged 45 and above, who may have lower levels of digital literacy. The results of the analyzed app-reported data suggest an overall high level of satisfaction with the digital therapy.

## 5. Limitations

The study, despite its rigorous design, is subject to several limitations that should be considered when interpreting the results. First, the lack of blinding of participants and clinicians could have introduced bias, as both groups were aware of the type of intervention being administered. However, since active treatment was provided to both study arms, positive expectations were likely to be present across both groups.

Second, the six-month intervention period, while effective for initial weight loss assessment, may not fully capture the long-term effects and sustainability of the outcomes. Plans to extend the study to evaluate the durability of these effects were hindered by high attrition rates following the intervention period. These high discontinuation rates were likely influenced by the unique challenges of the COVID-19 pandemic, which created significant obstacles for RCTs requiring in-person clinical assessments in hospital settings. This situation highlights the critical need for developing and implementing more effective strategies to enhance participant retention in randomized controlled trials focused on weight loss and obesity in order to ensure more comprehensive and reliable long-term results.

Furthermore, although the study targeted patients with obesity and glycemic disorders, the limited number of participants with type 2 diabetes, combined with the high prevalence of insulin resistance and near-optimal baseline HbA1c levels within the sample, limits the generalizability of the findings concerning glycemic control to a broader clinical population. This skewed participant profile means that the results may not fully capture the potential benefits or effectiveness of the interventions for all individuals with glycemic disorders. Consequently, the presented results suggest the effect of obesity management in diabetes prevention rather than treatment.

Additionally, there was an imbalance in the distribution of gender and educational levels among the study participants. Although statistical adjustments were made to account for these imbalances, they highlight a potential confounding factor that could influence the outcomes. Future studies should strive to recruit a more demographically balanced cohort or explicitly examine how these interventions might differentially impact various demographic groups.

## 6. Conclusions

In this non-inferiority trial, the comparable effectiveness of Vitadio, a digitally administered lifestyle intervention, and a traditional intensive weight management program has been demonstrated. The analysis of secondary outcomes further highlights the intervention’s role in promoting significant reductions in body fat and abdominal obesity without adversely affecting lean body mass, thus confirming its utility in supporting healthy weight loss. In addition, the intervention resulted in improved clinical outcomes for the participants who used the digital application. Favorable changes in lipid profiles and a decrease in HbA1c levels point to the broader cardiovascular and metabolic benefits of the examined treatment. These findings add to the recent body of evidence suggesting that digital health interventions can offer a viable alternative to conventional face-to-face approaches, potentially enhancing accessibility and addressing common barriers to obesity management, such as time constraints and costs.

## Figures and Tables

**Table 1:** Baseline demographic and clinical characteristics.

	Overall	IG	CG	*p*-Value
*n*	100	50	50	
Male	29 (29%)	16 (32%)	13 (26%)	0.66
Age	43.3 ± 9.5	43.3 ± 10.5	43.3 ± 8.4	0.99
*Education*				
Primary	5	3	2	
High school	75	33	42	0.12
College	20	14	6	
Type 2 diabetes	10	5	5	
Prediabetes	23	15	8	0.24
Insulin resistance	67	30	37	
*Diabetes medication* *				
None	23	15	8	
Metformin	7	3	4	
Sulfonylureas	1	1	0	0.6
Other	2	1	1	
*Anthropometric Parameters*				
Body weight (kg)	117.6 ± 20.9	117.5 ± 21	117.8 ± 21	0.94
BMI (kg/m^2^)	40.1 ± 6.1	40.5 ± 7.1	39.7 ± 5.1	0.51
Waist circumference (cm)	116.8 ± 14.7	118.1 ± 15.4	115.4 ± 14.0	0.36
Muscles mass (kg)	35.8 ± 7.5	35.8 ± 7.3	35.8 ± 7.9	1
Body fat (kg)	53.6 ± 13.4	53.0 ± 15.3	54.3 ± 11.3	0.62
*Glycemic Parameters*				
HbA1c (%)	5.6 ± 0.7	5.6 ± 0.7	5.5 ± 0.7	0.52
HOMA-IR	5.6 ± 4.0	6.3 ± 4.8	4.9 ± 2.8	0.06
FG (mmol/L)	5.7 ± 1.3	5.9 ± 1.5	5.6 ± 1.1	0.30
*Lipid Parameters*				
Total cholesterol (mmol/L)	4.9 ± 0.8	4.8 ± 0.9	4.9 ± 0.7	0.66
TAG (mmol/L)	2.1 ± 1.2	2.1 ± 0.9	2.0 ± 1.5	0.70
LDL (mmol/L)	2.8 ± 0.7	2.8 ± 0.8	2.9 ± 0.7	0.58
HDL (mmol/L)	1.1 ± 0.2	1.1 ± 0.2	1.2 ± 0.2	0.21
*Liver Enzymes*				
ALT (μkat/L)	0.8 ± 0.5	0.8 ± 0.6	0.7 ± 0.4	0.36
AST (μkat/L)	0.5 ± 0.3	0.5 ± 0.4	0.5 ± 0.3	0.77
GGT (μkat/L)	0.7 ± 0.6	0.7 ± 0.4	0.7 ± 0.8	0.57

* Type 2 diabetes and prediabetes participants only.

**Table 2:** Complete case analysis of weight change.

Group	*n*	Baseline	6 Months	Change	Cohen’s d (95% CI)	Results of LMM		3% Non-Inf. Margin (*p*-Value)
						Predictor	Coefficient	
IG	32	114.49 ± 19.5	107.25 ± 19.41	−7.25 ± 6.84 **	0.37 (0.24, 0.5)	6 months	−7.25 **	0.008
CG	28	116.75 ± 22.34	108.42 ± 23.9	−8.32 ± 8.22 **	0.35 (0.22, 0.49)	IG × 6 months	1.07	

** *p* < 0.01. The linear mixed-effects regression model includes data for three time-points—baseline and 3 and 6 months into the study. Only the outcomes at 6 months, as the final time-point of the study, are reported in the table. The model is adjusted for sex, age, education and diabetes progression level.

**Table 3:** ITT analysis of weight change.

Group	*n*	Baseline	6 Months	Change	Cohen’s d (95% CI)	Results of LMM		3% Non-Inf. Margin (*p*-Value)
						Predictor	Coefficient	
IG	50	117.46 ± 20.97	111.18 ± 19.85	−6.28 ± 7.25 **	0.3 (0.2, 0.4)	6 months	−6.28 **	0.03
CG	50	117.76 ± 20.98	112.55 ± 21.87	−5.21 ± 7.32 **	0.24 (0.14, 0.34)	IG × 6 months	−1.07	

** *p* < 0.01. The linear mixed-effects regression model includes data for three time-points—baseline and 3 and 6 months into the study. Only outcomes at 6 months, as the final time-point of the study, are reported in the table. The model is adjusted for sex, age, education and diabetes progression level.

**Table 4:** Changes in secondary outcome variables in the IG over 6 months (complete cases and intention-to-treat analysis).

Outcome Measure	Sample	*n*	Baseline	6 Months	Change	*p*-Value	Cohen’s d (95% CI)
BMI (kg/m^2^)	CC	32	39.44 ± 6.79	36.9 ± 6.88	−2.54 ± 2.09 **	<0.001	0.37 (0.26, 0.48)
	ITT	50	40.48 ± 7.07	38.38 ± 6.92	−2.1 ± 2.29 **	<0.001	0.3 (0.21, 0.39)
Waist circumference (cm)	CC	32	117.53 ± 15.51	111.41 ± 15.66	−6.12 ± 5.63 **	<0.001	0.39 (0.26, 0.53)
	ITT	50	118.12 ± 15.45	113 ± 14.53	−5.12 ± 6.29 **	<0.001	0.34 (0.22, 0.46)
Muscles mass (kg)	CC	32	35.48 ± 6.91	35.48 ± 7.09	0 ± 2.35	1.0	0 (−0.12, 0.12)
	ITT	50	35.78 ± 7.26	35.81 ± 7.18	0.02 ± 1.95	0.931	0 (−0.07, 0.08)
Body fat (kg)	CC	32	51.01 ± 14.84	44.04 ± 16.73	−6.97 ± 6.75 **	<0.001	0.42 (0.27, 0.58)
	ITT	50	52.95 ± 15.34	46.99 ± 16.38	−5.97 ± 6.74 **	<0.001	0.37 (0.25, 0.49)
HbA1c (%)	CC	23 ^1^	5.73 ± 0.78	5.35 ± 0.36	−0.38 ± 0.68 *	0.013	0.56 (0.11, 1.01)
	ITT	50	5.61 ± 0.7	5.43 ± 0.36	−0.18 ± 0.55 *	0.029	0.28 (0.03, 0.53)
HOMA-IR	CC	23 ^1^	5.88 ± 5.56	3.63 ± 3.38	−2.25 ± 6.44	0.107	0.49 (−0.13, 1.11)
	ITT	50	6.35 ± 4.83	5.27 ± 4.11	−1.07 ± 5.16	0.147	0.24 (−0.09, 0.57)
FG (mmol/L)	CC	24	6.1 ± 1.82	5.53 ± 0.84	−0.57 ± 1.58	0.092	0.36 (−0.07, 0.78)
	ITT	50	5.87 ± 1.5	5.59 ± 0.78	−0.28 ± 1.22	0.108	0.21 (−0.05, 0.47)
Total cholesterol (mmol/L)	CC	25	5.08 ± 0.94	4.73 ± 0.77	−0.34 ± 0.6 **	0.008	0.39 (0.11, 0.67)
	ITT	50	4.82 ± 0.88	4.66 ± 0.65	−0.16 ± 0.51 *	0.032	0.19 (0.02, 0.36)
TAG (mmol/L)	CC	25	2.25 ± 1.05	1.42 ± 0.52	−0.83 ± 0.98 **	<0.001	0.94 (0.4, 1.48)
	ITT	50	2.14 ± 0.87	1.64 ± 0.49	−0.5 ± 0.86 **	<0.001	0.68 (0.32, 1.05)
LDL (mmol/L)	CC	25	2.93 ± 0.8	2.86 ± 0.65	−0.08 ± 0.57	0.51	0.1 (−0.2, 0.41)
	ITT	50	2.79 ± 0.78	2.79 ± 0.55	0 ± 0.48	0.999	0 (−0.18, 0.18)
HDL (mmol/L)	CC	25	1.16 ± 0.25	1.24 ± 0.21	0.08 ± 0.15 *	0.017	0.31 (0.06, 0.56)
	ITT	50	1.1 ± 0.23	1.16 ± 0.2	0.06 ± 0.12 **	0.001	0.25 (0.11, 0.4)
ALT (μkat/L)	CC	22	0.7 ± 0.46	0.53 ± 0.2	−0.17 ± 0.42	0.069	0.45 (−0.05, 0.94)
	ITT	50	0.8 ± 0.64	0.68 ± 0.3	−0.13 ± 0.45	0.052	0.19 (0, 0.38)
AST (μkat/L)	CC	21 ^1^	0.52 ± 0.42	0.4 ± 0.11	−0.12 ± 0.43	0.219	0.37 (−0.24, 0.98)
	ITT	50	0.52 ± 0.36	0.45 ± 0.12	−0.07 ± 0.33	0.121	0.24 (−0.07, 0.55)
GGT (μkat/L)	CC	22	0.63 ± 0.35	0.55 ± 0.48	−0.07 ± 0.37	0.352	0.17 (−0.19, 0.54)
	ITT	50	0.66 ± 0.36	0.63 ± 0.46	−0.04 ± 0.27	0.326	0.09 (−0.09, 0.26)

^1^ Due to measurement error, HbA1c, HOMA-IR and AST values were not available for one of the participants; CC—complete cases; ITT—intention-to-treat; * *p* < 0.05; ** *p* < 0.01.

**Table 5:** Baseline characteristics of the IG participants who completed the study vs. those who dropped out.

Variables	Completers (*n* = 32)	Drop-Outs (*n* = 18)	*p*-Value
Weight	114.49 ± 19.5	122.72 ± 22.99	0.21
BMI	39.44 ± 6.79	42.32 ± 7.37	0.181
Waist	117.53 ± 15.51	119.17 ± 15.72	0.725
Age	46.12 ± 9.32	38.28 ± 10.94	0.015

## Data Availability

The data presented in this study are available to researchers who submit a methodologically sound proposal to the corresponding author, K.M. (katarina.moravcova@fnol.cz). To gain access to the data, proposers will be required to sign a data access agreement.

## Abbreviations

The following abbreviations are used in this manuscript:

ALT	Alanine aminotransferase
AST	Aspartate aminotransferase
BMI	Body mass index
CC	Complete case analysis
CG	Control group
CVD	Cardiovascular disease
DTx	Digital therapeutics
FG	Fasting glucose
GGT	Gamma-glutamyl transferase
HbA1c	Glycated hemoglobin
HDL-C	High-density lipoprotein cholesterol
HOMA-IR	Homeostatic Model Assessment for Insulin Resistance
IG	Intervention group
IR	Insulin resistance
ITT	Intention-to-treat analysis
LDL-C	Low-density lipoprotein cholesterol
LMCF	Last mean carried forward
MAR	Missing at random
OGTT	Oral glucose tolerance test
TAG	Triacylglycerol
